# Cubical and spherical directional array-based particle source detection with poisson statistics

**DOI:** 10.1038/s41598-025-92169-4

**Published:** 2025-04-04

**Authors:** Sahana Srikanth, Sanjeev Gurugopinath, Koshy George

**Affiliations:** 1 Department of Electronics and Communication Engineering, PES University, Bengaluru, 560085 India; 2 Department of Electronics and Communication Engineering, Gandhi Institute of Technology (GITAM), Bengaluru, 562103 India

**Keywords:** Engineering, Mathematics and computing

## Abstract

This paper investigates detecting a far-field dynamic particle source using cubical and spherical directional arrays. First, we present a discussion on likelihood ratio test (LRT), generalized LRT (GLRT) and truncated mean test (TMT) in case of a cubical array, considering scenarios with stationary and moving targets. Further, two underlying sub-cases are considered, including the sets of correlated and independent and identically distributed observations. Next, we consider the case of a spherical array where LRT, GLRT and TMT are discussed. In all cases, we present an analysis in terms of probabilities of detection and false-alarm for the associated hypothesis testing problems. Through simulations, we show that TMT outperforms the other existing tests in the literature, namely the mean difference test, the source intensity test, and the GLRT.

## Introduction

Detection and tracking of far-field particle sources is essential in diverse applications ranging from civilian to military. Some of the associated applications include nuclear activity detection^1,2^, mixed particle radiations^3^, wireless communication^4^, nuclear decommissioning^5^, astrophysics^6^, and robotics^7^. For the detection of particle sources, use of cubical and spherical directional detector arrays has been studied earlier^8,9^. Statistically, arrival of particle sources can be captured by random processes with highly localized-in-time discrete events. In particular, this is modeled and analyzed in the framework of Poisson processes, which naturally model the emission and measurement of particle sources^10^. However, most of the existing literature assumes that the observations under each hypotheses at the array are independent and identically distributed (i.i.d.) Poisson processes^10,11^. Liu et al. exploited the fact that in a cubical array, the presence of a source affects only the three faces of the array in its direction, to propose the mean-difference test (MDT)^9^. Extension of this test to a spherical array was carried out by modeling it as a polygonal detector array with a large number of faces. It was shown that when the signal-to-noise ratio (SNR) is low, the performance of MDT is superior to that of a generalized likelihood ratio test (GLRT)^12,13^. Another test termed as the source intensity test (SIT) was proposed by Bethel et al., whose performance is superior to that of MDT in terms of area under the receiver operating characteristics curve^14^. However, all the techniques mentioned above are based on the assumption that the source location is known or deterministic.

Moreover, it is also observed that the distribution of observations significantly deviate from the i.i.d. assumption, and closely follows a non-homogeneous Poisson process (NHPP)^15,16^. More recently, Romanchek et al. proposed a two-stage kernel-based approach was developed for detection of gamma rays in sparse and noise-dominated condition^17^. Cogswell et al. proposed a nuclear recoil detection based on color centres^18^. In this paper, we revisit the particle source detection problem using detector arrays that are directional. Our contributions can be summarized as below.

- With a cubical array, we derive the likelihood ratio test (LRT), GLRT and a truncated mean test (TMT) for the cases with NHPP and homogeneous Poisson process (HPP) observations with the source positions being deterministic but unknown, and random.
- We show that the TMT is optimal. In other words, LRT reduces to TMT in case of stationary sources. We find expressions for the probabilities of false-alarm and detection for TMT. In case of moving sources, we derive a test equivalent to TMT, termed as the truncated LRT (TLRT). Further, GLRT is shown to be helpful in scenarios where the underlying parameters are unknown.
- With a spherical array, we analyze the LRT, GLRT and TMT for stationary sources, and derive expressions for the probabilities of detection and false-alarm for TMT. Optimality of TMT is established even for this case.
- Finally, we use Monte Carlo simulations to validate the accuracy of our results and show that the performance of TMT is superior to MDT, SIT and GLRT.

A set of preliminary results with homogeneous Poisson processes by the authors of this paper is available in the literature^19^. Contributions of this work apart from the discussions in the conference version of this paper are as follows: We consider the particles striking at the cubic detector array to be an NHHP with log-linear intensity function, which better models real-world scenarios. The intensity takes the form of a function whose logarithmic is a function of parameters of the model. The log-linear models are flexible and have an ability to blend multiple parameters. Further, we extend our analysis to spherical detectors which is an extension of polygon detectors, for both non-homogeneous and homogeneous arrival rates. Additionally, we study the movement dynamics of the source on the detection performance, and derive the optimal test which is the TLRT. In each case, we validate the accuracy of our results through Monte Carlo simulations and show that truncated tests outperform other tests.

The remainder of the paper is organized as follows. The data model and directional arrays are defined in “Data model and directional arrays” section, which also gives an overview of the one-sided hypothesis testing problem along with the performance metrics. The cubical detector is examined in “Cubical arrays: detection of stationary sources” section. In Section, NHPP with varying intensity is defined, and GLRT is developed for a stationary source. For the case when the source location is known and random, a GLRT and truncated mean tests are developed in “Cubical arrays: detection of moving sources” section. Detection with spherical arrays is presented and analyzed in “inlinkSpherical arrays: detection of stationary sourcessec5” section, for varying and identically distributed intensities. In Section, the performance of a cubical array is analyzed for a moving source. Dataset generation and simulation results are presented and discussed in “Dataset generation and simulation results” section, and some concluding remarks are made in “Conclusion” section.

**Fig. 1:**
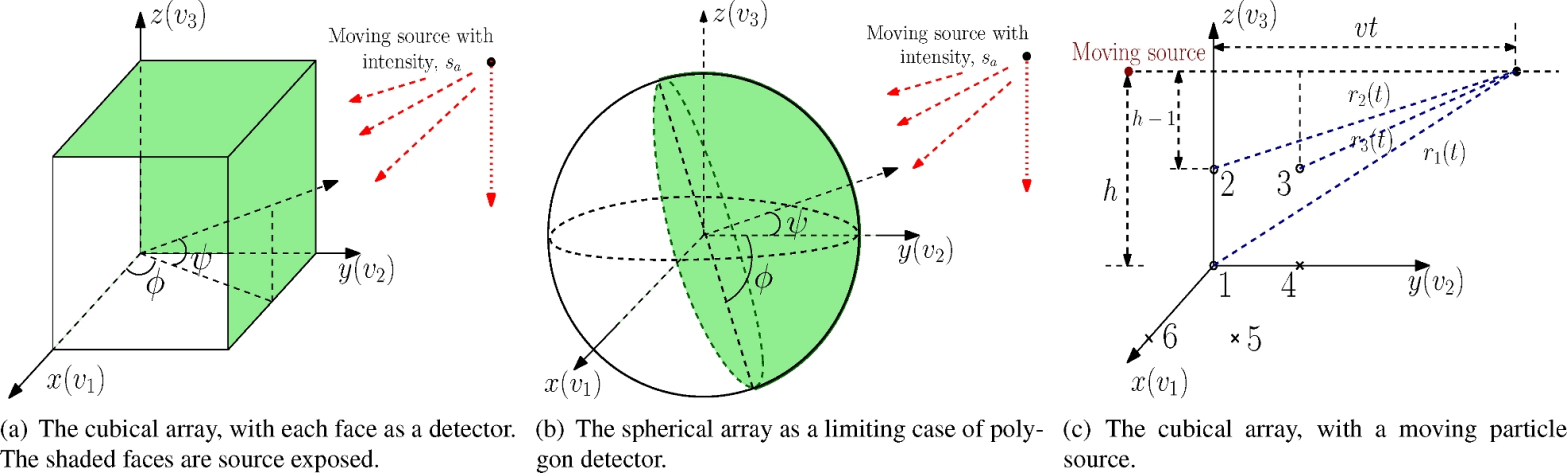
Cubical and spherical arrays, as special cases of polygon arrays.

## Data model and directional arrays

We consider the problem of detecting moving particle sources in the far field with projection-based arrays, as employing multiple sensors improves detection performance. Each face of this array is a detector. If the cross-sectional area of a face is $l_{cs}$, the number of particles striking it is proportionally related to it. The orientation vis-á-vis the source direction of each array detector is different. An assumption we make is as follows: the axes of the projection-based array and the reference coordinate system coincide. We further assume that the source’s constant velocity vector ${\textbf{v}}$ is parallel to the *y*-axis. Only a few detectors are exposed to a particle source whenever it is present, and we record the number of particles striking a sensor. We model the number of particles of the source *S* and the noise *n* as statistically independent Poisson processes. In the presence of a source, observations at each detector *i* is $\sigma _{i}(t) =S_{i}(t)+ n_{i}(t)$. The source detection can be simplified as the one-sided hypothesis testing problem:

1 $$\begin{aligned} {\mathcal {H}}_0: S_{i}(t) = 0, \nonumber \\ {\mathcal {H}}_1: S_{i}(t) > 0. \end{aligned}$$

In other words, this problem is summarized as follows. Given a time-realization vector of a Poisson process on a directional array, it is to be decided whether this vector has an intensity corresponding to noise-only observations or signal under noise observations. The above hypothesis testing problem can be solved using a test of the form

2 $$\begin{aligned} {\bar{T}}(\{S_{i}(t), i=1,\ldots , M\}) \overset{{\mathcal {H}}_1}{\underset{{\mathcal {H}}_0}{\gtrless }} \nu _{\text {\tiny T}}, \end{aligned}$$

where the sufficient statistic ${\bar{T}}(\{S_{i}(t), i=1,\ldots , M\})$ is a function of $S_1(t),\ldots ,S_M(t)$, and $\nu _{\text {\tiny T}} \in {\mathbb {R}}$ is the detection threshold. Performance of the test in (2) is evaluated in terms of the probability of false-alarm $P_{fa}$ and probability of detection $P_d$, which are defined as

3 $$\begin{aligned}&P_{fa} \triangleq P\{{\bar{T}}(\{S_{i}(t), i=1,\ldots , M\}) > \nu _{\text {\tiny T}} | {\mathcal {H}}_0\}, \end{aligned}$$

4 $$\begin{aligned}&P_{d} \triangleq P\{{\bar{T}}(\{S_{i}(t), i=1,\ldots , M\}) > \nu _{\text {\tiny T}} | {\mathcal {H}}_1\}, \end{aligned}$$

respectively. We employ the well-known Neyman–Pearson framework, which maximizes $P_d$ given a pre-defined $P_{fa} \in (0,1)$. In other words, $\nu _{\text {\tiny T}}$ is selected, based on a pre-defined $P_{fa} \in (0,1)$.

## Cubical arrays: detection of stationary sources

We begin with a cubical array with each face as a detector. It is a directional array, as the different faces are oriented differently relative to the direction of the source. As mentioned earlier, we assume a reference Cartesian coordinate system *C*(*x*, *y*, *z*) which coincides with the array’s axes. Suppose that the unit vectors corresponding to the positive directions of the three axes respectively are denoted ${\textbf{v}}_1$, ${\textbf{v}}_2$ and ${\textbf{v}}_3$^9^. Moreover, let the unit vectors corresponding to the negative directions of the three axes respectively be ${\textbf{v}}_4$, ${\textbf{v}}_5$ and ${\textbf{v}}_6$. Further, suppose that the source is initially located at (0, 0, *h*) units and moves at a constant velocity *v*, as shown in Fig. 1a. Here, *h* is the relative perpendicular distance between the source and *C*(*x*, *y*, *z*). In the presence of a source, the first three faces of the cubical array point to the particle source as depicted in Fig. 1a.

Each detector records the count of particles striking its surface. As mentioned earlier, we model the source particles and the noise as Poisson distributions statistically independent of each other. We denote by $\gamma (t)$ the expected number of source particles striking in a unit of time over a unit of area. In the absence of a particle source (under the hypothesis $H_0$), the sensors merely measure background noise with the expected count denoted by $\delta _n(t)$. The unit vector ${\textbf{d}} \triangleq \left[ d_1, d_2, d_3 \right] ^T = \left[ \cos \phi \cos \psi , \sin \phi \cos \psi , \sin \psi \right] ^T$ denotes the direction of the source, where the angles $\psi$ and $\phi$ respectively are the elevation and azimuth pointing towards ${\textbf{d}}$. Let the expected rate of source and noise particles be denoted $l_{cs} \gamma (t) d_i$ and $\delta _n(t) l_{cs}$, respectively. Here, $l_{cs} = l_{cs}({\textbf{d}} \cdot v_i)_+$, with $(g)_+ = \max {(g ,0)}$, is the cross-section of detector *i* in the direction of source. In the presence of a source, the observations at each detector *i* is $\sigma _{ij}(t) = l_{cs} \gamma _{ij}(t) d_i + n_{ij}(t)$, $1\le j \le N$, $1 \le i \le M$, where $\gamma _{ij}(t)$ and $n_{ij}(t)$, $t\in [0,T]$, respectively denote the intensities of source and background over *N* non-overlapping temporal measurements over *M* detectors. We define the parameter vector $$\varvec{\theta } (t) = \left[ \begin{array}{cccc} \phi&\psi&\gamma (t)&\delta _n(t) \end{array} \right] ^T$$ which is either known or unknown, depending on the situation at hand. Now, the source detection can be written as:

5 $$\begin{aligned} {\mathcal {H}}_0: \gamma (t) = 0, \nonumber \\ {\mathcal {H}}_1: \gamma (t) > 0. \end{aligned}$$

Here, ${\mathcal {H}}_0$ and ${\mathcal {H}}_1$ are the two hypotheses that correspond to the two scenarios: only noise is present and signal is present. Each detector *i* makes *N* non-overlapping observations over the time interval $t \in [0,T]$ and transmits to a fusion center, where they are processed using the Neyman–Pearson framework to decide the presence of a particle source. For ease of analysis, we initially consider the source stationary ($v=0$).

### Detection with correlated observations

We begin by considering the observations recorded at each detector to be an NHPP $\{N(t), t>0\}$. An NHPP is said to be log-linear if the intensity is defined by a function $\gamma (t) = e^{(A+Bt)}$, where *A* and *B* are unknown parameters. Consider an NHPP with events occurring at epochs $0< t_1< t_2< \cdots < t_n\le S$ with a mean value $\Lambda (t) = \int _{0}^{t} \gamma (u) du$ during the time interval (0, *T*). We observe that the arrival times of an NHPP are time-varying and the increments are non-stationary. The distribution of the inter-arrival times $s_1=t_1, s_2=t_2 - t_1, \ldots , s_n=t_n - t_{n-1}$ is given by Ref.^20^

$$\begin{aligned}&f(z) = \frac{1}{\gamma (T)} \int _{0}^{T-z} \gamma (y) \gamma (y + z) e^{-[\Lambda (y + z) - \Lambda (y)]} dy + \frac{\gamma (z) e^{-\Lambda (z)}}{\Lambda (T)}, \end{aligned}$$

where *f*(*z*) indicates the PDF of inter-arriving events. The problem in (5) reduces to

6 $$\begin{aligned}&{\mathcal {H}}_0: \sigma _{ij} = \delta _n(t_j) l_{cs}, \nonumber \\&{\mathcal {H}}_1: \sigma _{ij} = l_{cs} (e^{(A + Bt_j ) } + \delta _n(t_j))\mathbf {d_i} + \delta _n(t_j) l_{cs}, \end{aligned}$$

with $t_j$ determined by the PDF *f*(*z*). The unknown parameter vector can be expressed as ${\varvec{\theta }}(t) = [\phi ~ \psi ~ A ~ B ~ \delta _n]^T$. We consider here two situations. The parameter vector is deterministic and known or unknown in the first case, and the second corresponds to this vector being random. If the source’s location is known, it is quite straightforward to label the faces or detectors. We recall that the source-exposed faces of the cubic array are labeled 1, 2 and 3. Similarly, the respective opposite faces are labeled 4, 5 and 6. Secondly, when the source’s location is not known, we can estimate it based on sample means of opposing pairs of detectors, determined temporally^9^. After labeling the detectors arbitrarily, we first determine the sample means for a given *t*: $\widehat{\mathbf {\sigma }} \triangleq \left[ {\bar{\sigma }}_1 - {\bar{\sigma }}_4, {\bar{\sigma }}_2- {\bar{\sigma }}_5, {\bar{\sigma }}_3-{\bar{\sigma }}_6 \right] ^T$, where ${\bar{\sigma }}_i = \frac{1}{N} \sum _{j=1}^N \sigma _{ij}$, $1 \le i \le 6$. We then estimate the unit vector $\widehat{{\textbf{u}}} = {\widehat{\mathbf {\sigma }}}/ {\Vert \widehat{\mathbf {\sigma }}\Vert }$, where $\Vert \cdot \Vert$ is the 2-norm. With this estimated unit vector, $\widehat{{\textbf{u}}}$, the detectors are renamed. When the parameter $\varvec{\theta }$ is known perfectly, the optimal test is the likelihood ratio test (LRT). In contrast, when $\varvec{\theta }$ is unknown, LRT cannot be constructed. To this end, we determine the test statistic for NHPP based on the generalized LRT (GLRT). Given that each detector makes NHPP measurements in the presence of the source, the GLRT test at the fusion center is given by

7 $$\begin{aligned} {\bar{G}}_\sigma ^{cub} = \dfrac{ \underset{\theta , \delta _n}{\max }~L(\varvec{\sigma (t)}; {\varvec{\theta }},\varvec{\delta _n}, {\mathcal {H}}_1)}{\underset{\delta _n}{\max }~ L(\varvec{\sigma (t)};\varvec{\delta _n},{\mathcal {H}}_0)} \overset{{\mathcal {H}}_1}{\underset{{\mathcal {H}}_0}{\gtrless }} \nu _{\text {\tiny G}}^{cub}, \end{aligned}$$

where $L(\mathbf {\sigma (t)};\theta ,H_1)$ and $L(\mathbf {\sigma (t)};\delta _n,H_0)$ are the joint likelihood functions under each hypothesis, respectively, $\mathbf {\sigma (t)}$ = $[\sigma _{11}(t),\ldots ,\sigma _{1N}(t),\sigma _{21}(t),\ldots ,\sigma _{6N}(t)]^T$, $t \in [0,T]$ and $\nu _{\text {\tiny G}}^{cub}$ is the detection threshold chosen for a given probability of false-alarm $P_{fa}\in (0,1)$. To compute the likelihood ratio, we recall that all the 6*N* temporal measurements of the cubical detector are independent of each other. Therefore, the likelihood functions under $\mathcal {H_\text {0}}$ and $\mathcal {H_\text {1}}$ become

8 $$\begin{aligned}&L(\varvec{\sigma }; \varvec{\delta }, {\mathcal {H}}_0) = \prod _{i=1}^{6}\prod _{j=1}^N \delta _n(t_j) l_{cs} ~ \exp \left[ -\int _0^T \delta _n(t_j) l_{cs} dt \right] , \nonumber \\&L(\varvec{\sigma }; \varvec{\delta }, {\mathcal {A}}, {\mathcal {B}}, {\mathcal {H}}_1)=\prod _{i=1}^{3}\prod _{j=1}^N \left( e^{(A+Bt_j)}d_i + \delta _n(t_j) \right) l_{cs} \prod _{i=4}^{6}\prod _{j=1}^N \delta _n(t_j) l_{cs} \nonumber \\&\quad \times \exp \left[ -\int _0^T \delta _n(t_j) l_{cs} \, dt \right] \exp \left[ -\int _0^T \left( e^{(A+Bt_j)} d_i + \delta _n(t_j) \right) l_{cs} \, dt \right] , \end{aligned}$$

with exponents being the mean intensity value under each hypotheses. With $\delta _n(t)=\delta _n$, (7) reduces to

9 $$\begin{aligned}&{\bar{G}}_\sigma ^{cub} = \frac{N l_{cs}}{B} e^A \left( e^{B T - 1} \right) (\mathbf {d_1} + \mathbf {d_2} + \mathbf { d_3}) + \ln (l_{cs} e^A e^{B t_1} \mathbf {d_1} + \delta _n l_{cs}) + \cdots \nonumber \\&\quad + \ln (l_{cs} e^A e^{B t_N} \mathbf {d_1} \delta _n l_{cs}) + \ln (l_{cs} e^A e^{B t_1} \mathbf {d_2} + \delta _n l_{cs}) + \cdots + \ln (l_{cs} e^A e^{B t_N} \mathbf {d_3} + \delta _n l_{cs}). \end{aligned}$$

We do not consider an analysis on the detection performance using GLRT, since it is only used for performance comparison. Next, we extend our analysis of the NHPP process relaxing the time dependency between samples for complexity reduction and to develop a closed-form solution.

### Detection with IID observations

We consider a homogeneous Poisson measurement with an expected count of the particles being equal over the detectors with $\sigma (t) \equiv \sigma$. From the far-field assumption, $\sigma$ and $\delta _n$ are the same for all the detectors. Therefore, we define signal-to-noise ratio as $\sigma /\delta _n$. As mentioned earlier, we consider the case when parameter vector $${\varvec{\theta }} = \left[ \begin{array}{cccc} \phi&\psi&\sigma&\delta _n \end{array} \right] ^T$$, is random or deterministic. In the latter case, ${\varvec{\theta }}$ may not be known a priori.

#### Optimal truncated mean test

In this section, we ease our analysis of the cubical array’s test statistics based on the temporal sample mean of the observations. In the MDT, the first three detectors are exposed to particle source, and the remaining three are exposed only to noise^9^. We subtract the sample average of the measurements from the latter three detectors from the former three to mitigate the noise’s effect. If the source’s location is known a priori, or if it can be estimated by the method outlined earlier, the measurements from the latter three detectors are statistically redundant and can safely be ignored. The statistic of source-exposed detectors can be considered sufficient information to detect the presence of a source that does not require the knowledge of an estimate $\delta _n$. Moreover, if the source’s location is not known, we cannot label the detectors and hence cannot use the MDT. The proposed Truncated Mean Test (TMT) is based only on the source-exposed detectors’ statistic, constructed as

10 $$\begin{aligned} {\bar{T}}_\sigma ^{cub} \triangleq {\bar{\sigma }}_s = \frac{1}{3N} \sum _{i=1}^3 \sum _{j=1}^N \sigma _{ij} \overset{{\mathcal {H}}_1}{\underset{{\mathcal {H}}_0}{\gtrless }} \nu _{\text {\tiny T}}^{cub}. \end{aligned}$$

As noted before, ${\bar{\sigma }}_{s}$ is the temporal sample mean of the data from the three source-exposed detectors. The detection threshold $\nu _{\text {\tiny T}}^{cub}$ is optimally chosen based on the constraint on the false-alarm probability. The performance of TMT is described next.

##### Lemma 1

*Let the source’s location be known. Consider the binary hypothesis in* (6).* Then, the false-alarm and detection probabilities of TMT are respectively given by*

11 $$\begin{aligned}&P_{fa} = {\mathcal {Q}}\left( \dfrac{\nu _{\text {\tiny T}}^{cub} - \delta _n l_{cs}}{\sqrt{\frac{\delta _n l_{cs}}{3N}}}\right) , \end{aligned}$$

12 $$\begin{aligned}&P_d = {\mathcal {Q}} \left( \dfrac{{\mathcal {Q}}^{-1}(P_{fa}) \sqrt{\delta _n l_{cs}} - \mu l_{cs} {\bar{d}} \sqrt{3N}}{\sqrt{\mu l_{cs} {\bar{d}} + \delta _n l_{cs}}}\right) . \end{aligned}$$

*Here*, ${\bar{d}} =\frac{1}{3}\sum _{j=1}^3 d_j$* is proportional to the detector’s cross-section with reference to source’s direction. If the alignment of the cubical array is proper, the TMT is optimal in the Neyman–Pearson sense*.

##### Proof

See Appendix. $$\square$$

It is straightforward to see that the test statistic of TMT requires only an estimate of the sample mean of observations over the source-exposed detectors, which is quantified as ${\mathcal {O}}(MN)$, where *M* is the number of faces on a polygon array. In other words, TMT offers a computational advantage as it is linearly scalable with an increase in *M* and *N*. If the source’s location is either not known or random, we assume that the source is uniformly distributed on a three-dimensional ball around the cubic array with radius $\sqrt{3} l_{cs}/2$ with its center coinciding with the array. We can construct TMT by taking the temporal mean of the observations averaged over all the six faces of the detectors. In contrast, the MDT cannot be carried out as labeling of detectors are not possible. The following lemma describes the performance analysis for random source location.

##### Lemma 2

*Let the source’s location be unknown or random. Then, for the problem in* (5),* the false-alarm and detection probabilities of TMT are respectively given by*

13 $$\begin{aligned}&P_{fa} = {\mathcal {Q}}\left( \dfrac{\nu _{\text {\tiny T}}^{cub} - \delta _n l_{cs}}{\sqrt{\frac{\delta _n l_{cs}}{3N}}}\right) , \end{aligned}$$

14 $$\begin{aligned}&P_d = \int _{{\mathbb {R}}(S)} \left( \dfrac{{\mathcal {Q}}^{-1}(P_{fa}) \sqrt{\delta _n} l_{cs} - \sigma l_{cs} {\bar{d}}(s) \sqrt{3N}}{\sqrt{\sigma l_{cs} {\bar{d}}(s) + \delta _n l_{cs}}}\right) f_S(s) ds. \end{aligned}$$

*Here, the range of* *S is represented by* ${\mathbb {R}}(S)$* in polar form, and* ${\bar{d}} =\frac{1}{3}\sum _{j=1}^3 d_j$* is commensurate to the cross-section of all the detectors about the source’s direction*.

##### Proof

See Appendix . $$\square$$

We observe that the source’s location does not affect the false-alarm probability. Thus, the analysis is similar to that of a single stationary source. Moreover, the expression for the detection probability $P_d$ is averaged over $f_S(s)$.

#### Generalized likelihood ratio test

The hypothesis testing problem in (5) reduces to

15 $$\begin{aligned} \!\!\!\! & {\mathcal {H}}_0: \gamma _1 = \gamma _2 = \cdots = \gamma _6 = \delta _n l_{cs}, \nonumber \\ \!\!\!\! & {\mathcal {H}}_1: \gamma _1 = \sigma l_{cs} d_1 + \delta _n l_{cs}, \gamma _2 = \sigma l_{cs} d_2 + \delta _n l_{cs}, \gamma _3 = \sigma l_{cs} d_3 + \delta _n l_{cs}, \gamma _4 = \gamma _5 = \gamma _6 = \delta _n l_{cs}. \end{aligned}$$

The test statistic of GLRT (7) can be expressed as,

16 $$\begin{aligned} {\bar{G}}_\sigma ^{cub} = \dfrac{\max _{\theta , \delta _n} L(\varvec{\sigma }; {\varvec{\theta }},\varvec{\delta _n}, {\mathcal {H}}_1)}{\max _{\delta _n} L(\varvec{\sigma };\varvec{\delta _n},{\mathcal {H}}_0)} \overset{{\mathcal {H}}_1}{\underset{{\mathcal {H}}_0}{\gtrless }} \nu _{\text {\tiny G}}^{cub}, \end{aligned}$$

where $\mathbf {\sigma }$ = $[\sigma _{11},\ldots ,\sigma _{1N},\sigma _{21},\ldots ,\sigma _{6N}]^T$. Likelihood functions under ${\mathcal {H}}_0$ and ${\mathcal {H}}_1$ are:

$$\begin{aligned}&L(\varvec{\sigma }; \varvec{\delta }, {\mathcal {H}}_0) = \prod _{i=1}^M\prod _{j=1}^N \frac{{\bar{\sigma }}^{\sigma _{ij}} e^{-{\bar{\sigma }}}}{\sigma _{ij}!}, \nonumber \\&L(\varvec{\sigma }; \varvec{\theta }, {\mathcal {H}}_1) = \prod _{i=1}^{M/2}\prod _{j=1}^N \frac{{\bar{\sigma }}_s^{\sigma _{ij}} e^{-{\bar{\sigma }}_s}}{\sigma _{ij}!} \prod _{i=\frac{M}{2} +1}^{M}\prod _{j=1}^N \frac{{\bar{\sigma }}_n^{\sigma _{ij}}e^{-{\bar{\sigma }}_n}}{\sigma _{ij}!}. \end{aligned}$$

Here, ${\bar{\sigma }}$, ${\bar{\sigma }}_s$ and ${\bar{\sigma }}_n$ respectively are the averages determined over all the detectors — those that are exposed to the particle source and those that are not — computed temporally. The test in (16) reduces to,

17 $$\begin{aligned} \frac{1}{N} \ln {\bar{G}}_\sigma ^{cub} = \sum _1^3 \bar{\sigma _i} \ln \left( \frac{\bar{\sigma _i}}{{\bar{\sigma }}} \right) + 3{\bar{\sigma }}_n \ln \left( \frac{{\bar{\sigma }}_n}{{\bar{\sigma }}} \right) \overset{{\mathcal {H}}_1}{\underset{{\mathcal {H}}_0}{\gtrless }} g\left( \nu _{\text {\tiny G}}^{cub}\right) , \end{aligned}$$

where $g(\cdot )$ is a monotone function. We skip the derivations on $P_{fa}$ and $P_d$ for GLRT, since it is employed only for performance comparison with TMT.

## Cubical arrays: detection of moving sources

We consider the source is moving in a straight line at a constant velocity *v*, in the same direction as the *y*-axis. For ease of analysis, we assume that the source is at a relative distance *h* from the origin of the coordinate system as shown in Fig. 1c. We also assume that the faces of the cubical array act as point-detectors under the far-field assumption. It is evident that the coordinate points of these detectors are (0, 0), $(0,l_{cs})$ and $(l_{cs}, l_{cs})$ respectively. At a given instant *t*, we assume that each detector has access to the trajectory followed by the suspected source at all times in the interval [0, *T*]; otherwise, a suitable tracking algorithm is used. Let $r_i(t)$ denote the distance between the potential source and the detector *i*. We generalize the faces of the detectors as potential points as seen from the source. Without loss of generality, we denote $r_1(t)$, $r_2(t)$, $r_3(t)$ as the distances from the source-exposed detectors and $r_4(t)$, $r_5(t)$ and $r_6(t)$ denote the distances of noise-only detectors. These are given by

18 $$\begin{aligned}&r_1(t) = \sqrt{h^2+v^2t^2}, ~~~~~~~r_2(t) = \sqrt{(h- \sqrt{l_{cs}})^2+v^2t^2}, ~~~~~~~r_3(t) = \sqrt{(h- \sqrt{l_{cs}})^2+(vt- \sqrt{l_{cs}})^2}. \end{aligned}$$

Define $$J_s \triangleq \sum _{i=1}^{3} \int _0^T \mu _i(t)\,dt$$, where

19 $$\begin{aligned}&\int _0^T \mu _1(t)\,dt = \frac{\chi _s \alpha \tan ^{-1} \left( \frac{Tv}{hv} \right) }{hv}, ~~~ \int _0^T \mu _2(t)\,dt = \frac{\chi _s \alpha l_{cs} \tan ^{-1} \left( \frac{Tv}{h- \sqrt{l_{cs}}} \right) }{(h- \sqrt{l_{cs}})v }, \end{aligned}$$

20 $$\begin{aligned}&\int _0^T \mu _3(t)\, dt = \frac{2\chi _s \alpha \tan ^{-1} \left( \frac{2v^2T-2v}{\sqrt{4v^2(h^2-2h+2 \sqrt{l_{cs}})-4v^2}} \right) }{\sqrt{4v^2(h^2-2h+2 \sqrt{l_{cs}})-4v^2}}. \end{aligned}$$

The detectors record the number of particles striking on them at a time-varying rate denoted by $\sigma _i (t)$. The intensity at detector *i* due to the source (under ${\mathcal {H}}_1$) is given by $\sigma _s(t) = \chi _s \frac{\alpha }{r_j^2(t)}$, $1 \le j \le 6$. We denote the sensor’s cross-section coefficient by $\chi _a$ and the source’s activity by $\alpha$. The performance of source dynamics is described next.

### Lemma 3

*When the trajectory of the source is known, the optimal TLRT is*

21 $$\begin{aligned} L_\sigma \triangleq \prod _{i=1}^3 e^{-J_s} \prod _{j=1}^N \left( 1+\frac{\sigma _i(t_j(i))}{\delta _{ni}(t_j(i))}\right) \overset{{\mathcal {H}}_1}{\underset{{\mathcal {H}}_0}{\gtrless }} \nu _{\text {\tiny TLR}}^{cub}. \end{aligned}$$

*Here* $J_s$* is determined as in* (20). The $j^{\text {th}}$* jump time of the Poisson process corresponding to the* $i^{\text {th}}$* sensor is denoted* $t_j(i)$.* The optimal value of the detection threshold* $\nu _{\text {\tiny TLR}}^{cub}$* is chosen in accordance to the given probability* $P_{fa}$.

### Proof

See Appendix. $$\square$$

Taking logarithms on both sides of the test statistic of TLRT, it is observed that the computational complexity of this test is also ${\mathcal {O}}(MN)$, similar to that of TMT. Futher, it should be noted that evaluation of the detection threshold $\nu _{\text {\tiny TLR}}^{cub}$ is a one-time computation, which can be carried out offline.

## Spherical arrays: detection of stationary sources

In Sect., we discussed TMT and GLRT for cubical arrays. Here, this analysis is extended to spherical arrays. The spherical array is modeled as a polygonal detector array with many faces, i.e., $M \rightarrow \infty$, depicted in Fig. 1b. We consider that all the detectors are located on the surface of a sphere with a large number of small-sized detectors that completely cover the sphere’s surface. We assume that the sphere’s center coincides with the reference coordinate system. The detectors on the sphere are divided into two sets: source-exposed and noise-only detectors. We assume that the direction of the source is completely known.

### Detection with correlated observations

We again consider an NHPP with log-linear intensity function. We assume that the first half of detectors are always exposed to the source, with the other half having noise-only measurements. Assuming that the source-exposed and noise detectors originate from a point (under far-field assumption), the GLRT becomes,

22 $$\begin{aligned}&\ln {\bar{G}}_{\sigma (t)}^{sph} = -\frac{M}{2} \frac{N a}{B} e^A \left( e^{B T - 1} \right) \mathbf {{\bar{d}}} + \frac{M}{2N} \ln \left( l_{cs} e^A e^{B t_1} \mathbf {{\bar{d}}} + \delta _n l_{cs} \right) + \cdots + \ln \left( l_{cs} e^A e^{B t_N} \mathbf {{\bar{d}}} + \delta _n l_{cs}) \right) - \frac{M}{2}\ln \left( \delta _n l_{cs} \right) \!, \end{aligned}$$

where $\mathbf {{\bar{d}}}$ denote the direction of source-exposed detectors.

### Detection with IID observations

#### Optimal truncated mean test

We construct this test for spherical arrays based on the fact that the first *M*/2 detectors measurements are sufficient for detection. The test statistic can be constructed as

23 $$\begin{aligned} {\bar{T}}^{(\text {\tiny sph})}_s = \frac{2}{MN} \sum _{i=1}^{M/2} \sum _{j=1}^N \sigma _{ij} \overset{{\mathcal {H}}_1}{\underset{{\mathcal {H}}_0}{\gtrless }} \nu ^{\text {\tiny sph}}_{\text {\tiny TMT}}, \end{aligned}$$

where the threshold $\nu ^{\text {\tiny sph}}_{\text {\tiny T}}$ is fixed for a given $P_{fa} \in (0,1)$. We characterize the performance of TMT for spherical detectors in the following result.

##### Lemma 4

*Let the source’s location be deterministic either known or not. Then the probability of detection of TMT for spherical arrays, is given by*

24 $$\begin{aligned}&P_d = {\mathcal {Q}}\left( \frac{{\mathcal {Q}}^{-1}(P_{fa}) \sqrt{4 \delta _n} - \sigma \sqrt{\pi r^2 N M})}{\sqrt{2(\sigma +2\delta _n)}}\right) , \text { with } P_{fa} = {\mathcal {Q}}\left( \dfrac{\nu ^{\text {\tiny sph}}_{\text {\tiny TMT}} - 2 \pi \delta _n r^2}{\sqrt{\frac{4 \pi \delta _n r^2}{MN}}}\right) . \end{aligned}$$

##### Proof

See Appendix . $$\square$$

#### Generalized likelihood ratio test

Recall that the spherical array is an extension of a cubical array where $M\rightarrow \infty$. We construct this test by computing the joint likelihood functions under each hypothesis. We have the log-likelihood functions as,

$$\begin{aligned}&L(\varvec{\sigma }; \varvec{\theta }, {\mathcal {H}}_0)= \prod _{i=1}^M\prod _{j=1}^N \frac{{\bar{\sigma }}^{\sigma _{ij}} e^{-{\bar{\sigma }}}}{\sigma _{ij}!}, \\&L(\varvec{\sigma }; \varvec{\theta }, {\mathcal {H}}_1) =\prod _{i=1}^{\frac{M}{2}}\prod _{j=1}^N \frac{{\bar{\sigma }}_s^{\sigma _{ij}}e^{-{\bar{\sigma }}_s} \mathbf {{\bar{d}}}}{\sigma _{ij}!} \prod _{i=M/2 +1}^{M} \prod _{j=1}^N \frac{{\bar{\sigma }}_n^{\sigma _{ij}}e^{-{\bar{\sigma }}_n}}{\sigma _{ij}!}. \end{aligned}$$

Here, ${\bar{\sigma }}$, ${\bar{\sigma }}_s$ and ${\bar{\sigma }}_n$ respectively are the averages determined over all the detectors—those that are exposed to the particle source and those that are not—computed temporally. The test in (7) reduces to

25 $$\begin{aligned}&\ln {\bar{G}}_\sigma ^{sph} = \left( \sigma _{11} + \cdots + \sigma _{\frac{MN}{2}}\right) {\bar{\textbf{d}}} \ln \left( \frac{MN \bar{\sigma _s}}{2 {\bar{\sigma }}} \right) + \left( \sigma _{(M/2 + 1)1} + \cdots + \sigma _{MN} \right) \ln \left( \frac{MN \bar{\sigma _n}}{2 {\bar{\sigma }}} \right) - \frac{MN}{2} {\bar{\sigma }}_s + 2 {\bar{\sigma }} - {\bar{\sigma }}_n . \end{aligned}$$

In the next section, we explore the detection of a moving source using a cubical array.

**Fig. 2:**
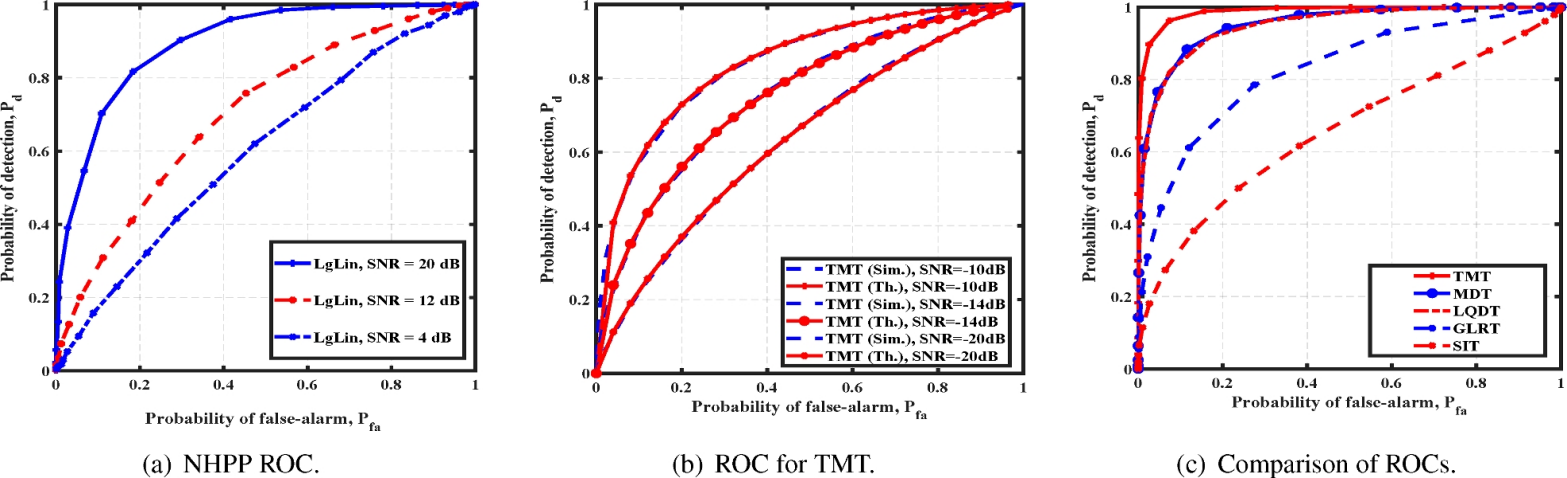
Cubical array: comparison of detection techniques for unknown but deterministic locations.

**Fig. 3:**
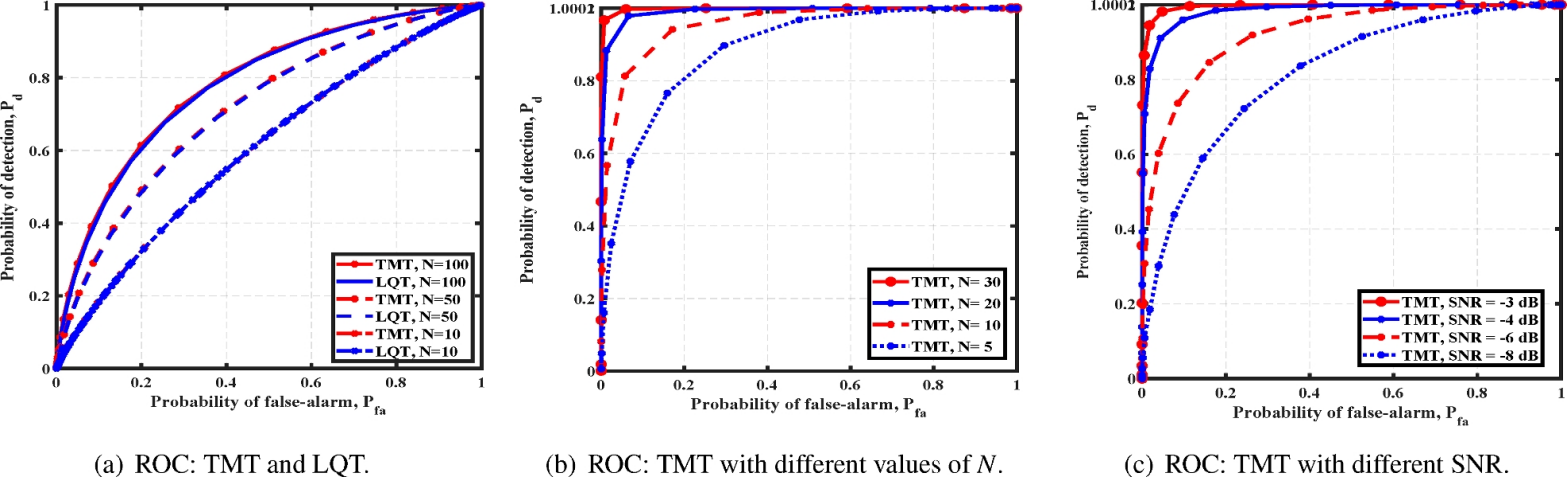
Cubical array: comparison of detection strategies with random locations.

## Dataset generation and simulation results

We first detail the methodology to generate datasets for our simulation study. Detector counts are generated as Poisson random variables with a given mean value, which corresponds to the expected count. Gaussian distributed random variables are added to these samples to simulate noise. Sampling parameters include the distances between source and each detector, azimuth and elevation angles, cross sections in the source direction, attenuating material (if any), source intensity, counting time and the number of random samples. Specific values of these parameters are mentioned later in this section. Method of generation of datapoints is similar to that described in Habrman et al. and Rahman et al., in three dimensions^21,22^. It is shown in Table I of Rahman et al. that employing this technique results in the statistics of the generated and measured datasets to be in good agreement with each other^22^. The generated datasets can be availed on request.

We first study the detection of a cubical array where the cross-sectional area of each face is $l_{cs}=1$. As mentioned earlier, we assume that the source’s direction is either known or estimated by the technique discussed a priori. We begin by considering a single stationary source at position $d =\frac{1}{\sqrt{(}3)}[1, 1, 1]^T$ from the reference coordinate system. We generate the arrival times of an NHPP using the expression *f*(*z*), with log-linear intensity function^23^. We compute optimal values of *A* and *B* based on the maximum likelihood function. For our simulation, we set $N =100$, $\delta _n =0.25$, and $P_{fa}=0.01$. For SNR, defined by $\frac{\sum A+Bt}{\eta l_{cs}}$, Fig. 2a shows the performance of GLRT. It is expected that the GLRT performs poorer than TMT, since it relies on the estimation of the underlying parameters, as opposed to TMT. The detection performance improves with SNR.

For the i.i.d. case, we consider the source intensity uniform and identically distributed over source exposed detectors. We validate the performance of TMT by comparing the theoretical and numerical analyses. From Fig. 2b, we observe a close match between the theoretically obtained plot and the one obtained via simulations, which validates our analysis for different SNRs. Further, it establishes the utility of our analysis for finite *N* even though it relies on employing CLT in the asymptotic regime of $N \rightarrow \infty$. Figure 2c, the TMT’s performance is compared with that of other detectors—GLRT, LQDT, MDT and SIT—for SNR = $-2$dB. As expected, TMT outperforms all other detectors, since it is optimal in the Neyman-Pearson sense. We also study the performance of TMT for different SNRs.

The source’s location is assumed to be uniformly distributed on a sphere with radius $\frac{\sqrt{3} d}{2}$ with $d=1$ being random whenever it is unknown and random. In Fig. 3a, the performance of TMT is compared with the linear quadratic test (LQT), a variant of TMT. In LQT, we include the quadratic and mean terms for source-exposed detectors to evaluate their effect on the performance. However, TMT performs similarly to LQT, concluding that the additional quadratic term is unnecessary. In Fig. 3b, we study the performance of TMT for an increase in *N*. We observe that as *N* increases, the detectors perform better. In Fig. 3c, we observe that $P_d$ improves with SNR.

**Fig. 4:**
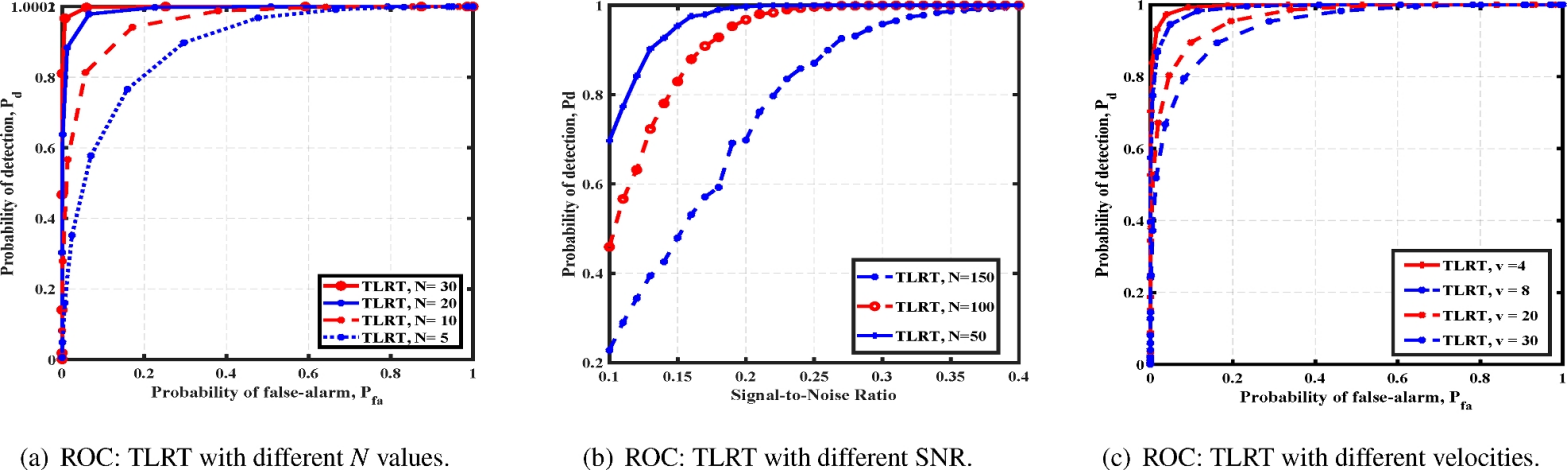
Cubical array: TLRT performances with a moving source.

Next, we consider a moving source and study the performance of a cubical array. We assume the source is moving at a distance $h = 10$ with a velocity $v = 8$. In Fig. 4a,b, we compare the performances with different values of *N* and SNR. As expected, we observe an improvement in performance with *N* and SNR. Further, TLRT exhibits better performance when the velocity of the source is decreased. The result in Fig. 4c indicates that the faster a source moves, the harder it is to detect.

**Fig. 5:**
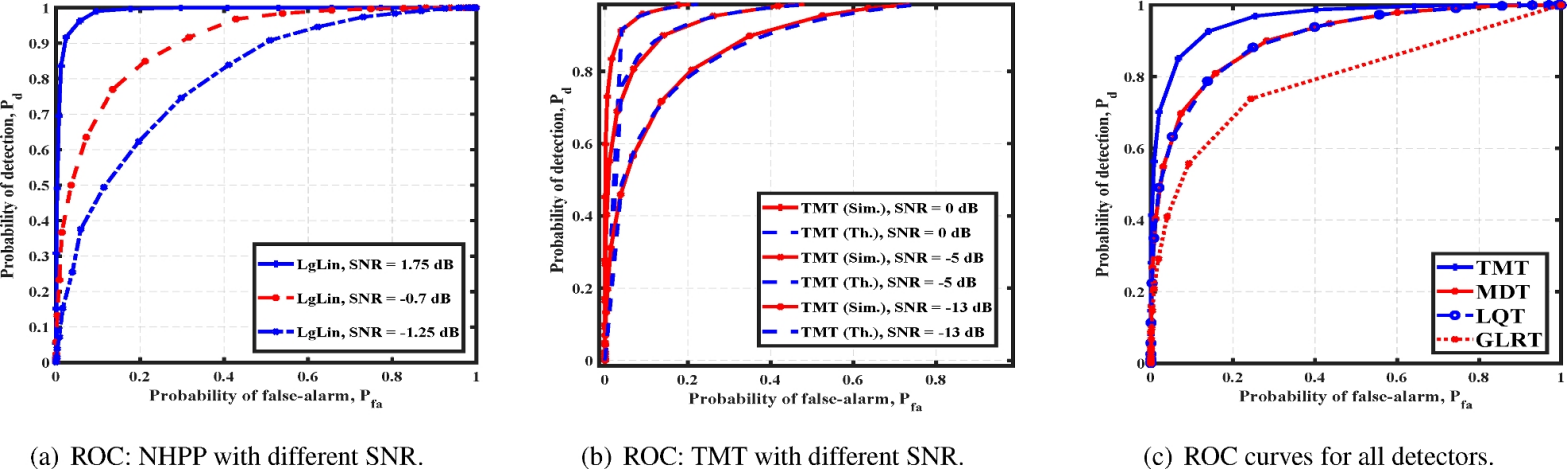
Spherical array: comparison of detection strategies for deterministic locations.

For a spherical array, we consider $M = 60$. We bring out similar simulation results for NHPP and i.i.d. cases. Figure 5a shows the performance of GLR for different SNRs. Figure 5b compares the simulation and theoretical analysis for TMT for different SNRs. In Fig. 5c, the performance of TMT is compared with all other detectors. Once again, the close match between the numerical and theoretical analysis validates our result. In either case, analysis of GLRT is complicated due to the non-Gaussian structure. We also observe that GLRT performs poorly at low SNRs and that TMT has proved superior. We observe that for a given $P_{fa}$, $P_d$ increases with an increase in SNR.

## Conclusion and future work

In this paper, we reconsidered the hypothesis testing problem of detecting a far-field moving particle source using directional detector arrays. We assumed time-varying and constant Poisson particle arrivals. We developed a novel test based on log-linear and fixed intensity functions. We developed GLRT and TMT when the source location is either known or random. We derived an asymptotic analysis of the performance of TMT. The detection performance of TMT is superior to other tests: MDT, GLRT, SIT and LQDT. We corroborate this by Monte Carlo simulations. Accordingly, TMT is optimal for detecting a stationary or moving particle.

This analysis can be extended to be focused on the finite number of observations regime to derive bounds on the performance of the detector. Further, the study on GLRT can be extended to estimate the unknown source location. Several assumptions were employed in this study, such as Poisson statistics for the particle source, known/deterministic source parameters such as location, and regime with large number of observations, to name a few. Such assumptions may not fully capture the real-world scenarios. Analysis of the particle source detection with polygon arrays in a more general framework with advanced techniques such as virtual arrays will be of significant interest in the field^24^. Towards this end, advanced detection techniques based on imaging, artificial intelligence or deep learning in a Bayesian framework can be employed^25^. Further, scenarios considered in this work can be extended to include losses of the particle source due to reflection, scattering, absorption, collision and orientation mismatch to study their effect on the detection performance. In addition to this, algorithms could be conceived to detect and estimate the real-time dynamics of the source. Utility of the proposed algorithms in real-world experimental scenarios for multiple applications is being investigated as a part of our future study.

## Data Availability

The datasets used and/or analysed during the current study available from the corresponding author on reasonable request.

## Appendix

### Proof of Lemma 1

Under the hypothesis ${\mathcal {H}}_0$, the observations $\sigma _{ij}$ is a Poisson random variable (PRV) with parameter $\delta _n l_{cs}$. Therefore, the statistic, $\sum _{i=1}^3 \sum _{j=1}^N \sigma _{ij}$ is a PRV with parameter $3N\delta _n l_{cs}$. As *N* grows unbounded, we invoke the central limit theorem (CLT). Accordingly, the sample mean ${\bar{\sigma }}_{s}$ follows a Gaussian distribution, i. e., ${\bar{T}}_\sigma ^{cub} \sim {\mathcal {N}}\left( \delta _n l_{cs}, \frac{\delta _n l_{cs}}{3N}\right) .$ Now, under $H_1$, the observations $\sigma _{ij}$ is a PRV with parameter $\sigma l_{cs} d_i + \delta _n l_{cs}$. It can easily be shown that the statistic

26 $$\begin{aligned} \sum _{i=1}^3 \sum _{j=1}^N \sigma _{ij} \sim \texttt{Poi}(3N\sigma l_{cs} {\bar{d}} + 3 N \delta _n l_{cs}). \end{aligned}$$

Again, invoking CLT for large *N*, the sample mean ${\bar{\sigma }}_{s}$ follows Gaussian distribution, i. e.,

27 $$\begin{aligned} {\bar{T}}_\sigma ^{cub} \sim {\mathcal {N}}\left( \sigma l_{cs} {\bar{d}} + \delta _n l_{cs},~ \frac{\sigma l_{cs} {\bar{d}}}{3N} + \frac{\delta _n l_{cs}}{3N} \right) . \end{aligned}$$

The probabilities $P_{fa}$ and $P_d$ can be determined by considering the corresponding tail probabilities of ${\bar{T}}_{\sigma }^{cub}$.

### Proof of Lemma 2

Consider a sphere around the cubic array centered at the array axis with radius $\sqrt{3} l_{cs}/2$. We assume that the source location is distributed on this sphere. Further, we assume, without loss of generality, that the source’s probability density function is uniform, denoted $f_S(s)$, $S \sim {\mathcal {U}}\left( -\frac{\sqrt{3}l_{cs}}{2}, \frac{\sqrt{3}l_{cs}}{2} \right)$, in polar coordinates. The azimuth angle lie in the range $(0,2 \pi )$. Similarly, the elevation angle lie in the range $(-\pi /2,\pi /2)$. It follows that the test statistic TMT reduces to the temporal sample mean of the observations over all time and across all detectors. The test for the case of unknown location is given as

28 $$\begin{aligned} {\bar{T}}_\sigma ^{cub} \triangleq {\bar{\sigma }} = \frac{1}{6N} \sum _{i=1}^6 \sum _{j=1}^N \sigma _{ij} \overset{{\mathcal {H}}_1}{\underset{{\mathcal {H}}_0}{\gtrless }} \nu ^{cub}_{\text {\tiny T}}, \end{aligned}$$

where $\nu ^{cub}_{\text {\tiny T}}$ is the optimal detection threshold.

### Proof of Lemma 3

Each detector computes the distance $r_i$ to the source. Further, it records those instants of time $t_j(i)$, $j\ge 1$, when the particle counts are registered. At the end of each interval of duration *T*, the fusion center combines the statistics of each sensor to calculate the likelihood ratio given by

29 $$\begin{aligned} L_\sigma (i) \triangleq e^{-\int _0^T \sigma _i(t)\,dt} \prod _{j=1}^N\left( 1+\frac{\sigma _i(t_j(i))}{\delta _{ni}(t_j(i))}\right) . \end{aligned}$$

With only first three detectors exposed to the potential source with the average count denoted by $J_s$, the statistic

30 $$\begin{aligned} {\mathcal {L}}_{\sigma } = \prod _{i=1}^{3} e^{-J_s} \prod _{j=1}^N\left( 1+\frac{\sigma _i(t_j(i))}{\delta _{ni}(t_j(i))}\right) , \end{aligned}$$

is compared with threshold to determine the probabilities of detection and false alarm.

### Proof of Lemma 4

Under ${\mathcal {H}}_0$, the statistic $\sum _{i=1}^{M/2}\sum _{j=1}^N \sigma _{ij}$ is a PRV with parameter $({MN/2}) 2\pi r^2 \delta _n$. Then asymptotically, by CLT it follows that the statistic of truncated mean test for spherical arrays has a Gaussian distribution, as $T_\sigma ^{sph} \sim {\mathcal {N}}\left( 2 \pi r^2 \delta _n,~ \frac{4 \pi r^2 \delta _n}{MN} \right)$. Now, under ${\mathcal {H}}_1$, $\sigma _{ij}$ is a PRV with expected rate $({MN/2}) \pi r^2 (\sigma + 2 \delta _n)$. As before, we employ CLT for large values of *N*. Thus, $\mathcal {{\bar{\sigma }}}$ is distributed as

31 $$\begin{aligned} T_\sigma ^{sph} \sim {\mathcal {N}}\left( \pi r^2 (\sigma + 2\delta _n), ~ \frac{ \pi r^2 (\sigma + 2 \delta _n)}{MN} \right) . \end{aligned}$$

Again, the probabilities $P_{fa}$ and $P_d$ are evaluated with the corresponding tail probabilities of TMT.
